# Intrapartum Antibiotic Prophylaxis and Early-Onset Neonatal Sepsis Patterns

**DOI:** 10.1080/10647440300025525

**Published:** 2003

**Authors:** Rodney K. Edwards, Whitney E. Jamie, Donald Sterner, Susan Gentry, Kathy Counts, Patrick Duff

**Affiliations:** 1 Department of Obstetrics and Gynecology Division of Maternal-Fetal Medicine University of Florida College of Medicine Gainesville FL USA; 2 Department of Information Services Shands Hospital at the University of Florida PO Box 100294 1600 SW Archer Road Gainesville FL 32610–0294 USA

## Abstract

*Objective:* To compare the relative effects of intrapartum antibiotic prophylaxis regimens on patterns of early-onset neonatal sepsis.

*Methods:* We performed an historical cohort study of 17 187 infants born at our center from September 1993 to February 2000. A risk-based strategy was employed prior to July 1996 and a screening-based strategy was utilized thereafter. Ampicillin was utilized prior to March 1995 and penicillin was used thereafter.

*Results:* There were 75 cases of neonatal sepsis, 34 (4.10/1000) in the risk-based era and 41 (4.63/1000) in the screening-based era (*p* = 0.62). There were fewer ampicillin-resistant isolates during the risk-based than the screening-based era (32 versus 61%; *p* = 0.014). The only significant change in organism-specific sepsis rates was an increase in the rate of infection caused by coagulase-negative staphylococci in the screening-based era (0.36 versus 1.46/1000; *p* = 0.018), but 75% of infants infected with these organisms were not exposed to ß-lactam antibiotics within 72 h prior to delivery. For the risk- and screening-based eras, respectively, the rates of Gram-negative sepsis (1.21 versus 1.46/1000; *p* = 0.65) and the proportions of Gram-negative pathogens that were ampicillin-resistant (70 versus 77%; *p* = 1.0) were similar. The drug employed for prophylaxis did not appear to affect the pattern of sepsis cases.

*Conclusion:*  In our patient population, coagulase-negative staphylococci have become the most common cause of early-onset neonatal sepsis. The cause of this shift in pathogen prevalence is uncertain and seemingly unrelated to intrapartum antibiotic exposure.


*Infect Dis Obstet Gynecol 2003;11:221–226*

### Intrapartum antibiotic prophylaxis and early-onset neonatal sepsis patterns

**Rodney K. Edwards[1], Whitney E. Jamie[1], Donald Sterner[2], Susan Gentry[1], Kathy Counts[2] and Patrick Duff[1]**

[1]*Department of Obstetrics and Gynecology, Division of Maternal-Fetal Medicine, University of Florida College of Medicine, Gainesville, FL*
[2]*Department of Information Services, Shands Hospital at the University of Florida, Gainesville, FL*

***Objective:*** To compare the relative effects of intrapartum antibiotic prophylaxis regimens on patterns of early-onset neonatal sepsis.
***Methods:*** We performed an historical cohort study of 17 187 infants born at our center from September 1993 to February 2000. A risk-based strategy was employed prior to July 1996 and a screening-based strategy was utilized thereafter. Ampicillin was utilized prior to March 1995 and penicillin was used thereafter.
***Results:*** There were 75 cases of neonatal sepsis, 34 (4.10/1000) in the risk-based era and 41 (4.63/1000) in the screening-based era ($p = 0.62$). There were fewer ampicillin-resistant isolates during the risk-based than the screening-based era (32 versus 61%; $p = 0.014$). The only significant change in organism-specific sepsis rates was an increase in the rate of infection caused by coagulase-negative staphylococci in the screening-based era (0.36 versus 1.46/1000; $p = 0.018$), but 75% of infants infected with these organisms were not exposed to β-lactam antibiotics within 72 h prior to delivery. For the risk- and screening-based eras, respectively, the rates of Gram-negative sepsis (1.21 versus 1.46/1000; $p = 0.65$) and the proportions of Gram-negative pathogens that were ampicillin-resistant (70 versus 77%; $p = 1.0$) were similar. The drug employed for prophylaxis did not appear to affect the pattern of sepsis cases.
***Conclusion:*** In our patient population, coagulase-negative staphylococci have become the most common cause of early-onset neonatal sepsis. The cause of this shift in pathogen prevalence is uncertain and seemingly unrelated to intrapartum antibiotic exposure.

Key words: GROUP B STREPTOCOCCUS; ANTIBIOTIC RESISTANCE; PREGNANCY; STAPHYLOCOCCI

Guidelines for the prevention of perinatal group B streptococcal disease using intrapartum antibiotic prophylaxis were issued by the Centers for Disease Control and Prevention in 1996[1]. These guidelines were revised in 2002[2]. Both versions of the guidelines recommend penicillin as the preferred agent for intrapartum antibiotic prophylaxis and refer to ampicillin as an 'acceptable alternative'. Penicillin was recommended preferentially due to its narrower spectrum and presumably, its theoretical advantage in causing less selection of β-lactam antibiotic-resistant organisms. The more recent guidelines preferentially recommend use of a screening-based method (over a risk-based method) of selecting women to receive intrapartum antibiotic prophylaxis[2]. This recommendation is based on a cohort study that demonstrated that routine screening for

Correspondence to: Rodney K. Edwards, MD, MS, University of Florida, Department of Obstetrics and Gynecology, PO Box 100294, 1600 SW Archer Road, Gainesville, FL 32610–0294, USA. Email: edwardsr@obgyn.ufl.edu

© 2003 The Parthenon Publishing Group 

group B streptococcus (GBS) during pregnancy prevented more cases of early-onset neonatal GBS disease (relative risk 0.46)[3].

Retrospective and observational data from multiple studies suggest that intrapartum antibiotic prophylaxis with ampicillin increases both the incidence of early-onset neonatal infection with Gram-negative bacteria of the *Enterobacteriaceae* family and the proportion of these organisms that are resistant to ampicillin[4–7]. However, this association has not been consistently demonstrated in all studies[8,9]. We are unaware of any data that have been published that implicate intrapartum antibiotic prophylaxis with penicillin in increasing the likelihood of early-onset neonatal infection with Gram-negative organisms. Unfortunately, nor are there any published data that refute that such an association exists.

Early-onset neonatal infections with ampicillin-resistant *Enterobacteriaceae* result in an increased mortality rate compared with infections with ampicillin-sensitive bacteria[4–6]. Since the screening-based strategy results in more widespread use of antibiotics, one concern regarding the most recent recommendation for intrapartum antibiotic prophylaxis is the potential for increasing the rates of sepsis caused by more virulent pathogens. The objectives of this study were to evaluate the relative effects of intrapartum antibiotic prophylaxis regimens (risk- or screening-based approach) on neonatal sepsis patterns and to compare the relative effects of penicillin and ampicillin on these sepsis patterns.

### SUBJECTS AND METHODS

We performed an historical cohort analysis of live-born infants delivered at Shands Hospital at the University of Florida from September 1, 1993 to February 25, 2000. A risk-based strategy for intrapartum antibiotic prophylaxis was employed prior to July 1, 1996 and a screening-based strategy was utilized thereafter. Ampicillin was used for prophylaxis prior to March 1, 1995 and penicillin was used thereafter.

Infants were included in the cohort if they had a positive blood culture during the first 7 days of life. Infants born elsewhere and then transferred to our center for neonatal care were excluded, as were infants whose cultures were of organisms generally considered to be contaminants (e.g. *Corynebacterium* sp., *Bacillus* sp.). Cases with coagulase-negative staphylococci were included only if the infant received anti-staphylococcal antibiotics for at least 5 days. This stipulation was included in an attempt to objectively eliminate cases in which blood cultures positive for coagulase-negative staphylococci clinically were considered to represent contaminated cultures.

Cases of positive blood cultures within the first 7 days of life were identified from the laboratory database for Shands Hospital at the University of Florida that is maintained by the hospital's Department of Information Services. Both aerobic and anaerobic blood cultures were included. During the entire study period, blood cultures were obtained in aerobic and anaerobic resin culture bottles and a pediatric blood culture bottle and analyzed using an automated system (Bactec, BD, Sparks, MD). Antibiotic susceptibility testing was done using the MicroScan minimum inhibitory concentration breakpoint determination for both Gram-positive and Gram-negative bacteria (Dade Behring, Inc., W. Sacramento, CA). The medical records of these infants then were reviewed to determine whether the subject met the criteria for inclusion in the cohort. For those subjects meeting criteria for inclusion, their records were abstracted for demographic, clinical and outcome data. The mothers of the infants included as subjects were identified from the database maintained by the Division of Maternal–Fetal Medicine in the Department of Obstetrics and Gynecology and the medical records of these women were reviewed and abstracted for demographic data and peripartum clinical information. The University of Florida Health Center Institutional Review Board approved the study.

The primary outcome variable was the proportion of isolates causing early-onset neonatal sepsis cases that were resistant to ampicillin. Isolates with intermediate susceptibility were considered resistant. Secondary outcomes included total and organism-specific rates of early-onset sepsis, susceptibility of Gram-negative isolates to ampicillin, and neonatal mortality. We also evaluated the effect of exposure to any β-lactam antibiotic within 72 h prior to delivery on neonatal sepsis patterns. All statistical tests were two-tailed and used an $\alpha$ of 0.05. The uncorrected chi-square and Fisher's exact tests were used to analyze categorical data. The unpaired Student's *t*-test and Mann–Whitney *U* were utilized for continuous data, as appropriate. To detect a 100% increase in rate of ampicillin resistance from 30% during the risk-based strategy to 60% during the screening-based strategy, 84 subjects were needed ($\alpha = 0.05$; $1\text{-}\beta = 0.80$).

### RESULTS

During the study period, 17 187 infants were delivered at our center: 8287 during the risk-based era and 8900 during the screening-based era. Seventy-five infants met the criteria for inclusion in this cohort study. Table 1 presents the demographic data for subjects in the risk- and screening-based era groups.

Shown in Table 2 are the total and organism-specific sepsis rates for each era. In the risk- and screening-based groups there were ten and 12 coagulase-negative staphylococci isolates, respectively, that were considered to be contaminants, and these cases were eliminated from further analysis.

Considering all cases of early-onset neonatal sepsis collectively, there was a lower proportion of isolates resistant to ampicillin during the risk- than the screening-based era (32 versus 61%; $p = 0.014$). This increase in the proportion of ampicillin-resistant cases was primarily due to the increase in the proportion of cases caused by coagulase-negative staphylococci (Table 2). The proportion of Gram-negative isolates that were resistant to ampicillin was similar between groups (70 compared with 77% for the risk- and screening-based eras, respectively; $p = 1.0$). There was a trend toward increased mortality during the screening-based era, but this difference did not reach statistical significance (0 versus 9.8%; $p = 0.13$).

Considering the entire cohort collectively, 17 of 28 (61%) total isolates from subjects exposed to $\beta$-lactam antibiotics within 72 h prior to delivery were resistant to ampicillin, while 20 of 47 (43%) isolates from subjects not exposed were resistant to ampicillin ($p = 0.13$). Of cases of *E. coli* sepsis, eight of the 11 (73%) subjects exposed to $\beta$-lactam antibiotics had ampicillin-resistant isolates, while three of the four (75%) unexposed subjects had ampicillin-resistant isolates ($p = 1.0$). Similarly, of cases of sepsis caused by any Gram-negative organism, 11 of the 14 (79%) subjects exposed to $\beta$-lactam antibiotics had ampicillin-resistant isolates, while six of the nine (67%) unexposed subjects had ampicillin-resistant isolates ($p = 0.64$). No isolates of GBS, and all isolates of coagulase-negative staphylococci, were resistant to ampicillin. During each era, 75% of cases of early-onset sepsis caused by coagulase-negative staphylococci occurred in

**Table 1** Demographic data for early-onset sepsis cases occurring in the risk- and screening-based eras

| *Variable* | *Risk* (*n* = 34) | *Screening* (*n* = 41) | *p value* |
|---|---|---|---|
| Gestational age (weeks) | 34.1 ± 5.3 | 32.6 ± 5.8 | 0.23 |
| Race | | | 0.68 |
| White | 14 (41) | 19 (46) | |
| Black | 18 (53) | 18 (46) | |
| Other | 2 (6) | 4 (10) | |
| Birth weight (grams) | 2375 ± 1118 | 2019 ± 1236 | 0.20 |
| Neonatal gender | | | |
| Male | 18 (53) | 26 (63) | 0.36 |
| Delivery route | | | 0.30 |
| Vaginal | 19 (56) | 18 (44) | |
| Cesarean | 15 (44) | 23 (56) | |

Data are presented as *n* (%) or mean ± standard deviation

**Table 2** Total and organism-specific early-onset sepsis rates for the risk- and screening-based eras

| *Rates of sepsis* | *Risk* (*n* = 34) | *Screening* (*n* = 41) | *p value* |
|---|---|---|---|
| Overall | 34 (4.10) | 41 (4.63) | 0.62 |
| Group B streptococci | 14 (1.69) | 9 (1.01) | 0.22 |
| *Escherichia coli* | 7 (0.84) | 8 (0.90) | 0.91 |
| Total Gram-negative | 10 (1.21) | 13 (1.46) | 0.65 |
| Coagulase-negative staphylococci | 3 (0.36) | 13 (1.46) | 0.018 |
| Other | 7 (0.84) | 6 (0.67) | 0.67 |

Data are presented as *n* (rate per 1000)

infants that were not exposed to β-lactam antibiotics within 72 h prior to delivery.

The majority of infants with early-onset sepsis during each era were born preterm. During the risk-based era, 18 (53%) of the 34 septic infants were born prior to 37 weeks, compared with 29 (71%) of the 41 septic infants born during the screening-based era ($p = 0.11$). Of cases of sepsis caused by coagulase-negative staphylococci, all three during the risk-based era and 11 of 13 during the screening-based era occurred in preterm infants. Presented in Table 3 are the total and organism-specific sepsis rates for each era, stratified by term versus preterm delivery.

During the risk-based era, 4912 deliveries occurred during the time period when ampicillin was utilized and 3375 deliveries occurred when penicillin was utilized. As noted in the larger (risk versus screen) group comparisons, the ampicillin and penicillin groups were similar with regard to distributions of race, gender and delivery route and mean gestational age at delivery and birth weight.

Shown in Table 4 are the total and organism-specific sepsis rates for each era, ampicillin or penicillin, during the risk-based strategy era. In the ampicillin and penicillin groups, respectively, there were six and four coagulase-negative staphylococci isolates that were considered to be contaminants. During the ampicillin and penicillin eras, respectively, six of 17 (35%) and five of 17 (29%) isolates were resistant to ampicillin. All four of the Gram-negative isolates in the ampicillin group and four of the six isolates in the penicillin group were resistant to ampicillin ($p = 0.47$).

In the entire cohort, only three infants were infected with bacterial isolates that had intermediate susceptibility to ampicillin. One of these infants was delivered during the risk-based era and two of them were delivered during the screening-based era. Only one of these infants (delivered during the screening-based era) was exposed to β-lactam antibiotics within 72 h prior to delivery. As described in the Subjects and Methods section, these isolates were considered to be resistant to ampicillin. If they had been considered susceptible, the results of the statistical comparisons would not be substantially different.

**Table 3** Total and organism-specific early-onset sepsis rates for the risk- and screening-based eras, stratified by term (≥ 37 weeks gestation) or preterm delivery

| *Rates of sepsis* | *Risk* (*n* = 34) | *Screening* (*n* = 41) | *p value* |
|---|---|---|---|
| Overall | | | |
| Term | 16 (2.45) | 12 (1.75) | 0.45 |
| Preterm | 18 (10.22) | 29 (14.30) | 0.30 |
| Group B streptococci | | | |
| Term | 12 (1.84) | 6 (0.87) | 0.16 |
| Preterm | 2 (1.14) | 3 (1.48) | 1.0 |
| *Escherichia coli* | | | |
| Term | 3 (0.46) | 4 (0.58) | 1.0 |
| Preterm | 4 (2.27) | 4 (1.97) | 1.0 |
| Total Gram-negative | | | |
| Term | 3 (0.46) | 4 (0.58) | 1.0 |
| Preterm | 7 (3.98) | 9 (4.44) | 1.0 |
| Coagulase-negative staphylococci | | | |
| Term | 0 (0.00) | 2 (0.29) | 0.50 |
| Preterm | 3 (1.70) | 11 (5.42) | 0.07 |
| Other | | | |
| Term | 1 (0.15) | 0 (0.00) | 0.49 |
| Preterm | 6 (3.41) | 6 (2.96) | 1.0 |

Data are presented as *n* (rate per 1000)

**Table 4** Total and organism-specific early-onset sepsis rates for the ampicillin and penicillin portions of the risk-based era

| *Rates of sepsis* | *Ampicillin* (*n* = 17) | *Penicillin* (*n* = 17) | *p value* |
|---|---|---|---|
| Overall | 17 (3.46) | 17 (5.04) | 0.27 |
| Group B streptococci | 7 (1.43) | 7 (2.07) | 0.48 |
| *Escherichia coli* | 3 (0.61) | 4 (1.19) | 0.45 |
| Total Gram-negative | 4 (0.81) | 6 (1.78) | 0.33 |
| Coagulase-negative staphylococci | 2 (0.41) | 1 (0.30) | 1.0 |
| Other | 4 (0.81) | 3 (0.89) | 1.0 |

Data are presented as *n* (rate per 1000)

### DISCUSSION

Intrapartum antibiotic prophylaxis is recommended, not as a permanent solution to perinatal GBS disease, but as a temporizing measure until other methods of control are available and can be implemented (e.g. vaccination)[1,2]. Widespread adoption of intrapartum antibiotic prophylaxis resulted in a 65% decrease in the incidence of early-onset neonatal GBS infection from 1993 to 1998[10]. In addition, use of a screening-based, as opposed to a risk-based approach, has now been associated with fewer maternal infections[11] and fewer cases of neonatal GBS infections[3].

However, a prior study from our institution reported that use of a screening-based approach results in more women receiving antibiotics in labor[11]. In some studies, more widespread use of intrapartum antibiotic prophylaxis has been associated with increased rates of early-onset neonatal infection with ampicillin-resistant Gram-negative bacteria[4–7]. This change in the pattern of neonatal sepsis is important, since the mortality rate for infants with ampicillin-resistant Gram-negative infections is approximately 50%, while the rate for infants with GBS sepsis is in the range of 5%[5,6]. Furthermore, our recent clinical trial lends credence to the biological plausibility that intrapartum antibiotic prophylaxis (with either ampicillin or penicillin) may increase the likelihood of neonatal infection with ampicillin-resistant Gram-negative organisms[12].

In contrast to our hypothesis, we did not show an increase in the proportion of early-onset neonatal sepsis cases caused by ampicillin-resistant Gram-negative organisms coincident with the adoption of a screening-based method for selecting candidates for intrapartum antibiotic prophylaxis. Instead, we demonstrated that coagulase-negative staphylococci (previously the third most common cause) have become the most common cause of early-onset neonatal infections at our center. Our results do show a significant increase in the proportion of pathogens that are resistant to ampicillin, coincident with using the screening-based regimen. However, the most striking (and the only statistically significant) change in pathogen-specific infection rates was this increase in the incidence of cases caused by coagulase-negative staphylococci.

Furthermore, three-quarters of the infants who developed infections with coagulase-negative staphylococci were not exposed to any β-lactam antibiotic within the 72 h prior to delivery. This fact argues against intrapartum antibiotic prophylaxis having had a major role in the shift in sepsis patterns having occurred. Certainly, the reasons for any such shift are multiple and complex.

We observed a trend toward a higher proportion of ampicillin-resistant organisms causing early-onset sepsis in those infants who had been exposed to any β-lactam antibiotic during the 72 h prior to delivery, regardless of the purpose for which the antibiotic was administered. This exposure was due, in many cases, to antibiotic therapy aimed at prolonging the latency period in the setting of preterm premature rupture of the membranes. Prior to March 1, 1998, our regimen for preterm premature rupture of the membranes was intravenous ticarcillin-clavulanate for 48 h followed by oral amoxicillin-clavulanate for another 5 days. Since March 1, 1998, our regimen has been a single oral dose of azithromycin plus intravenous ampicillin for 48 h followed by oral amoxicillin for another 5 days. Perhaps the pattern of antibiotic use throughout pregnancy, or in the population in general, may have contributed to the change in the proportion of ampicillin-resistant organisms causing early-onset sepsis. Almost half of the women who receive intrapartum antibiotic prophylaxis at our center receive additional antibiotics during their antepartum course[12].

However, the shift in prevalence of pathogens causing early-onset neonatal sepsis may have more to do with factors other than antibiotic prescribing practices. In the early part of the twentieth century, group A streptococci were the most frequent cause of perinatal infections. Later, *E. coli* was the most frequently isolated pathogen. By the 1970s, GBS became the most frequent cause of early-onset neonatal sepsis[13]. The reason(s) for this shifting pattern in pathogens causing early-onset neonatal sepsis have not been explained satisfactorily. Perhaps we will see (and are already seeing at our center) coagulase-negative staphylococci emerge as the most important pathogen in cases of early-onset neonatal infections.

As in our recent clinical trial[12], we documented no advantage of penicillin over ampicillin in this study. The subgroup analysis comparing the effects of these two antibiotics in this study is rather small and sweeping conclusions would be inappropriate. However, we continue to believe that the choice of agent for intrapartum antibiotic prophylaxis should be based on such factors as cost, availability and patient tolerability, rather than any theoretical advantage of penicillin over ampicillin.

Since intrapartum antibiotic prophylaxis is only considered a temporary and imperfect solution to early-onset neonatal GBS disease, ongoing surveillance of patterns of sepsis cases remains an important endeavor. Efforts aimed at optimizing the process for selecting candidates for prophylaxis and at solutions other than antibiotic prophylaxis should continue to receive our attention.

### REFERENCES

1. Centers for Disease Control and Prevention. Prevention of perinatal group B streptococcal disease: a public health perspective. *MMWR* 1996;45:1–24
2. Centers for Disease Control and Prevention. Prevention of perinatal group B streptococcal disease. *MMWR* 2002;51:1–22.
3. Schrag SJ, Zell ER, Lynfield R, *et al*. A population-based comparison of strategies to prevent early-onset group B streptococcal disease in neonates. *N Engl J Med* 2002;347:233–9
4. Towers CV, Carr MH, Padilla G, Asrat T. Potential consequences of widespread antepartal use of ampicillin. *Am J Obstet Gynecol* 1998;179: 879–83
5. Joseph TA, Pyati SP, Jacobs N. Neonatal early-onset *Escherichia coli* disease: the effect of intrapartum ampicillin. *Arch Pediatr Adolesc Med* 1998;152:35–40
6. Levine EM, Ghai V, Barton JJ, Strom CM. Intrapartum antibiotic prophylaxis increases the incidence of Gram-negative neonatal sepsis. *Infect Dis Obstet Gynecol* 1999;7:210–13
7. O'Reilly G, Hitti J, Brock B, Watts DH, Gill P, Benedetti T. Gram-negative sepsis among low-birth-weight infants: infection rates before and after initiation of group B streptococcus prophylaxis. *Am J Obstet Gynecol* 2001;184:S14
8. Chen KT, Tuomala RE, Cohen AP, Eichenwald EC, Lieberman E. No increase in rates of early-onset neonatal sepsis by non-group B *Streptococcus* or ampicillin-resistant organisms. *Am J Obstet Gynecol* 2001;185:854–8
9. Baltimore RS, Huie SM, Meek JI, Schuchat A, O'Brien KL. Early-onset neonatal sepsis in the era of group B streptococcal prevention. *Pediatrics* 2001;108:1094–8
10. Schrag SJ, Zywicki S, Farley MM, *et al*. Group B streptococcal disease in the era of intrapartum antibiotic prophylaxis, 1993–1998. *N Engl J Med* 2000;342:15–20
11. Locksmith GJ, Clark P, Duff P. Maternal and neonatal infection rates with three different protocols for prevention of group B streptococcal disease. *Am J Obstet Gynecol* 1999;180:416–22
12. Edwards RK, Clark P, Sistrom CL, Duff P. Intrapartum antibiotic prophylaxis. 1: relative effects of recommended antibiotics on Gram-negative pathogens. *Obstet Gynecol* 2002;100:534–9
13. Schuchat A. Group B streptococcal disease: from trials and tribulations to triumph and trepidation. *Clin Infect Dis* 2001;33:751–6

RECEIVED 05/12/03; ACCEPTED 08/24/03
